# Effective criteria in the public-private partnership in developing countries to apply the sustainable development goals: GAN-based decision support system for the renewable electrical system, case study Syria

**DOI:** 10.1016/j.heliyon.2023.e21422

**Published:** 2023-10-25

**Authors:** Vladimír Krepl, Ghaeth Fandi, Mohammad Rehabi, Safwan Ghanem, Fayez Jrad, Zdenek Muller, Luboš Smutka, Jan Kyncl, Melkior Urbanus, Soliman Fandie, Inna Čábelková, Josef Tlustý

**Affiliations:** a Department of Economics, Faculty of Economics and Management, Czech University of Life Sciences Prague, Kamýcká 961/129, Prague 6, 165 00 Prague, Czech Republic; b Centre of International Rural Development Studies (CIRDS), Faculty of Economics and Management, Czech University of Life Sciences Prague, Kamýcká 961/129, Prague 6, 165 00 Prague, Czech Republic; c Department of Sustainable Technologies, Faculty of Tropical AgriSciences, Czech University of Life Sciences Prague, Kamýcká 961/129, Prague 6, 165 00 Prague, Czech Republic; d Department of Electrical Power Engineering, Faculty of Electrical Engineering, Czech Technical University in Prague, Technická 2, Prague 6, 166 27 Prague, Czech Republic; e Department of Trade and Finance, Faculty of Economics and Management, Czech University of Life Sciences Prague, Kamýcká 961/129, Prague 6, 165 00 Prague, Czech Republic; f Department of Construction Engineering and Management, Faculty of Civil Engineering, Tishreen University, Latakia, Syria; g Department of Health Care Disciplines and Population Protection, Faculty of Biomedical Engineering, Czech Technical University in Prague, Sportovců 2311, 272 01 Kladno, Czech Republic

**Keywords:** Effective criteria, Private-public partnership, Electrical system, Sustainable energy, Generative adversarial neural networks, Sustainable development goals

## Abstract

The cost of generating electricity in developing countries surpasses the government's ability to sustain it, necessitating the involvement of the private sector in this service provision through public-private partnerships (PPPs) contracts. In Syria, the electricity system has been highly susceptible to damage as a result of the ongoing crisis, leading to frequent and prolonged blackouts. This research focuses on addressing the need for a comprehensive system that aids decision-making for PPPs contracts in the country. By employing a combination of studies, reports, and interviews with domain experts, significant general and exclusive factors that guide decision-makers in PPPs contracts are identified and organized into questionnaires. These questionnaires are then filled out by professionals engaged in PPPs contracts. The collected data is analyzed and validated using SPSS software. However, due to insufficient data collected, generative adversarial neural networks (GAN) are utilized to enhance the research data. Additionally, Expert Choice and the analytic hierarchy process are employed to calculate weights for each factor. Remarkably, the calculated weights for both general and exclusive factors align with real-life strategies. General factors primarily address the financial and commercial considerations associated with PPPs, while exclusive factors primarily focus on the operational aspects of the electrical power system. These factors are arranged in descending order of effectiveness, enabling stakeholders to determine whether the private sector should be engaged in the project or if it should remain within the public sector's purview. The proposed system has demonstrated its reliability and can serve as a promising starting point for PPPs contracts.

## 1 Introduction

Joint international construction contracts are among the hot research topics in both economic and political investigations. These contracts play an important role in developing countries to meet their desired growth goals. Goal No. 17 in [1], [2], “Partnerships to achieve the goals”, is the preferred goal to be achieved. Therefore, economic countries have already started to legislate legal and economic strategies. Those strategies aim to facilitate the process of upcoming economic changes in terms of partnership change [3], [4]. The backbone of the new strategies is opening the door for nongovernmental economic organizations and associations to increase direct investment in leading economic projects. Subsequently, involving those entities in the leading-actor role of achieving the development [5].

The partnership is not dividing the work between the public and private sectors, but also changing the development philosophy [6], [7]. Moreover, rebuilding the role of the government and its responsibilities of managing the national economic affairs by following strategies. The focus of those strategies is to transfer the private sector to the partner zone in all economic and social fields [8], [9].

The public-private partnerships (PPPs) decision cannot be taken randomly, and it must be made on a scientific basis. That includes the effective criteria for PPPs contacts to decrease the mistakes that cannot be avoided after the decision [1], [2]. Those criteria have been investigated in many researches and it has been proven that criteria highly differ from one country to another depending on legal, economic, social, and political conditions. Therefore, framing the correct PPPs relies on country-wise factors and conditions [10].

The electrical sector is an attractive field for investing by private entities because of its demand propositions with the population. That assures continuous market in long term. Moreover, the electrical sector is always on the decision table of governments in developing countries, especially for countries suffering from a crisis like Syria.

In this article, the criteria that affect the decision-making about PPPs are deeply investigated. The electrical power system in Syria is chosen as an example of a public sector that can be reconstructed using PPP contracts in a developing country suffering from a long-term crisis that almost destroyed that sector completely. Given the high cost of such a project and the importance to have a stable power system, there is a necessity to make a decision on the possibility of cooperation with the private sector. The criteria have been organized in a questionnaire that has been distributed among and filled by domain experts. SPSS software tool has been used to verify the completeness of responses. The number of valid responses was low and it might have led to wrong outcomes thus, there was a need to obtain more data. Considering that many domain experts were not reachable or refused to respond, it was beneficial to use the generative adversarial network (GAN) to enrich the dataset.

Syria has substantial untapped potential for renewable energy development. By focusing on the renewable electrical system, this case study aims to explore how public-private partnerships can play a crucial role in harnessing Syria's renewable energy resources for sustainable development. This objective is aligned with the global sustainable development goals, particularly in terms of reducing reliance on fossil fuels, mitigating the impact of climate change, and promoting access to clean and affordable energy sources.

There is a need to define the criteria for making those decisions because they represent big steps in developing countries. Those facts motivate authors to propose a comprehensive decision support system considering the following:

1. General criteria of the PPPs
2. Exclusive criteria of the PPPs related to the electrical system structures
3. Data enriching using GAN model
4. Obtaining additional sustainable solutions that enable people in charge to make decisions and specify the goals based on scientific bases

The literature review is presented in the next section. The analytical statistical methodology is detailed in Section 3. Research tools and the used software are described in Section 4. Section 5 discusses the experimental results. The conclusion and the scope of future improvements are given in Section 6.

## 2 Literature review

The number of countries that depend on partnerships has increased internationally. That enables the private sector to provide the basis of infrastructure and the required services such as roads, airports, railways, ports, and energy. Those services have high revenue thus they are the most nominated ones to be applied on a partnership basis. The partnership has also a great effect on the government side because it exposes the necessity for governments to ensure the partnership's efficiency. This efficiency is essential to provide high levels of services considering important factors such as the logical framework, the executive procedure and choosing the companies, and the contracts' commitments that the partnership depends on [5]. While partnerships have become the pre-eminent model for RET programs it was shown how multi-actor partnerships came to be seen as a means for improving the sustainability of development assistance for renewable energy. Therefore, investing in building power plants will lead to growth and development, which is clearly visible in the economic part. Since the government sectors are unable to provide these investments, we turned to the private sector to invest in this field in a way that interests both parties [11].

The statics pointed out that the international increment in electrical demand reached 6% in the period 2014-2020 and will reach 5.5% in 2020-2025, and 7% in 2025-2030 [12]. It is clear from these statistics that government budgets will be at stake with the only possible solution is the contribution of the private sector. The importance of PPPs is explained in a report issued by the United Nations Economic and Social Commission for West Asia report in 2017. The report confirmed the significance of PPPs for achieving sustainable development goals, especially goal No. 17, through the necessity of establishing a renewable and vital partnership. The partnership should be based on mutual values, vision, and goals that connect the civil community with government efforts. The report summarized the required foundations as the existence of a legislative framework, security and political stability, the existence of a national reference, exemption from some fees and taxes, availability of necessary data and information, involvement of civil society groups, and benefit from previous experiences.

The e8 and World Energy Council presented a group of recommendations in their report in 2011 [13]. Those recommendations resulted from a questionnaire about the international partnership in April 2010. That report suggested that nations should have renewable energy services and decrease international energy by about 40% by 2030. Therefore, the partnership with the private sector should be established, and the partnership process should be developed and encouraged enough by highlighting its crucial role. The report also confirmed that defining partnership contracts is the base for reaching the desired transformation.

British High Commission in its special furnish program in 2012 presented a report for partnership contracts in India as an example of developing countries. The report mentioned that the PPPs are important tools to facilitate private sector investment, the importance of private sector investment, and the need to participate with the private sector in providing public services including water and electricity [14].

The partnership is important to be operated in the energy sector for developing countries that have been exposed to the electrical sector destroyed including power plants, substations, power transition lines, and distribution substation centers. Syria is an example that is considered among the worst scenario within developing countries. The electrical sector has been significantly damaged and, consequently, Syria transferred from being an electricity-exported country with more than 6000 MW daily power production from local resources by 99% before the crisis in 2011; to a country that can provide 28% at the maximum of its needs [15]. Syrian public association for producing electricity reported that the losses in the electricity sector reached 2.728 billion Dollars until 29.Sep.2017. Therefore, it is hard for the Syrian government to pay a huge amount of money to invest in the electrical sector and rebuilt the whole system again. Thus it is required to give opportunities to the private sector to participate in the development process there.

The announcement of the Syrian strategies for the partnership has been launched at the PPPs conference convened by the Syrian-British Association on 30.Oct.2009. Syria showed that there is a need to invest 11 billion Dollars to meet the increased electrical demand by 2020 [16]. The needed acts have been legislated and discussed at later conferences to facilitate the partnership, especially after the crises that affected the infrastructure from 2011 until that date. Act No. 5 in 2016 concerns the partnership contracts and that law has explained partnership as a contractual relationship between private and public sides for a period of time in which, the private side provides public service through investment [17].

There are criteria that specify the possibility and feasibility to implement a project based on PPPs. Those criteria are to be considered as results of growth goals and economic and social needs in Syria. In [18] criteria for PPPs are deemed to be the legal environment and the organizing structure, the rhyming transparency environment of the partnership projects, the economic and social environment, the cultural and public environment, and the human resources.

There are success factors for the partnership that can switch the competitor private sector in Syria into a strong investor partner in the growth and its sustainability. Those factors are outlined in [19] as the legal factor, the economic factor, the management factor, the operational factor, the social factor, and the organizing factor.

The increasing demand for electricity during 2010-2020 posed a challenge to the limited budget of the public sector. On the other hand, oil revenue decreased during that period in parallel with the increasing burden of spending on essential social services such as education, and health. As a response to the power challenge, two power plants have been agreed to be established. The first one is decided to be in Suwaydia, and the other one is to be located in the free zone in Adra. The capacity is chosen to be 450 MW for each. Given the limited budget of the public sector and the crisis consequences, the aforementioned projects are realized not to be implemented only by the public sector but also by the participation of the private sector. Therefore, law No. 5 was legislated in 2016 to detail the regulations for PPPs contracts [15].

Energy loss and climate change should be also considered when thinking about enhancing electrical systems. The energy sector should decrease the number of factories that release CO2 to create a pollution-free environment [20]. The international hope is to prevent more climate change by considering renewable energy as a main resource to achieve the social, economic, and environmental goals for human beings [21], [22], [23], [24]. Renewable energy projects in developing countries almost do not exist, even though those countries, including Syria, have renewable energy resources like wind, solar, and biomass energy. Those resources can be used to strengthen production and ease the distribution of energy, and that can be done through PPPs scheme.

Accessing data resources in Syria is challenging due to both legal restrictions and strategic considerations. Studies in literature have addressed this issue by using data-enriching techniques. Generative adversarial neural networks (GANs) have shown great potential for enriching datasets by generating synthetic data samples that resemble the original data. GANs can be used in various domains such as healthcare, finance, and computer vision to generate synthetic data points that can expand the size and diversity of the dataset. Researchers have developed different variants of GANs, such as improved Wasserstein GAN with gradient penalties [25], Progressive GAN, and StyleGAN, which have achieved impressive results in generating realistic and high-quality synthetic data. These GAN-based approaches have the potential to address data scarcity issues, enhance data augmentation, and improve the performance of various machine learning models by providing more representative and comprehensive datasets. Furthermore, recent research [26] has explored the integration of GANs with transfer learning techniques to further enhance the data enrichment process. Overall, GANs have become an essential tool for dataset enrichment in machine learning and are expected to continue evolving and improving in the coming years.

## 3 Methodology

The analytical statistical methodology has been made by defining the criteria and factors that have an immediate effect on PPPs based on prior experiences and studies. In addition to experts' opinions taken from individuals who are in charge and involved in PPPs collaboration. The collected criteria are further classified into two groups:

1. General criteria that directly affect the partnership regardless of the type of project. This criteria group specifies the success or failure of PPPs in both developed and developing countries. Respondents were chosen among specialist people in contract management, engineering management, business administration, and economics. Furthermore, the academic people from the National Institute of Administration that was established based on the French-Syrian agreement. The number of the sample with verified data is 40 people.
2. Exclusive criteria that directly affect the project according to the desired service provided by the project. These services can be in power, transportation, infrastructure, communications, etc. This group of criteria is considered to be the second phase after taking the participatory decision based on the general factors. It was vital to target managers and electrical power specialists jointly. Therefore, the questionnaire was directed to the chief executive officers in the electrical power in Syria in many important units, generate, transport, and distribute. The number of valid samples for this questionnaire was 20 respondents.

In this research, the exclusive factors are related to the electrical energy structures. The priority of each factor is represented by a given weight that clarifies the importance of each.

The study in this work depends on two types of data:

• The preliminary data, that have been obtained from the practical part of the research through questionnaires. Domain experts duly filled out the questionnaires in connection with their experiences. Thereafter, answers are extracted, processed, and analyzed using SPSS.
• Secondary data, that have been obtained from books, reports, research, and published literature. This helps to indicate the structure of the requested questionnaires in their earlier versions.

GAN is used to to produce a wide variety of data, sampling from different places in the target distribution as the collected data via questionnaires. Arranging the criteria to each other through weights calculated through the application AHP (Analytic Hierarchy Process), where the Expert Choice program was used to find out the order of Criteria from most important to least important.

Ethical approval was obtained from participants in the questionnaire to keep the collected data for research purposes.

## 4 Research tools

This section details the used research tools. The questionnaire methodology for collecting data is used in this work. Therefore, the questionnaire design is highlighted. Moreover, the collected data from the questionnaire have not been enough to be analyzed due to less participation in a such questionnaire, and to authors' will not include inconsistent data. Therefore, data have been enriched using GAN which is one of the cutting-edge technologies used for enriching data.

### 4.1 Questionnaire design

Two questionnaires have been designed after studying the effective criteria of PPPs contracts and after interviewing domain experts. The first questionnaire has been created for the general factors, and the second one has questions related to private factors that are related to laws in Syria. Respondent has to choose one of five options that are very important, important, neutral, not important, and not important at all.

#### 4.1.1 General factors

The first part contains financial and commercial factors to start the PPPs project. Five questions are introduced in this part to include as much as possible economic sides. The level of questions starts from the economical stability and the economic feasibility study to the duration of the investment and its revenue.

The second part deals with the political factors to start the PPPs project. Three questions are presented to cover the importance of political conditions in terms of stability and partnership continuity. This part also covers the governmental support of the projects and their suitability for the current stage of growth.

The third part covers the legal factors to start the PPPs project. Three questions are introduced to detail the contract scheme between both parties. That includes a clear guide to contracting, flexibility, and the division of risks between those parties.

The fourth part is the technical factors to start the PPPs project. Three questions are used to highlight the importance of the technical factors starting from the experience of both sectors in build-operate-transfer (B.O.T.) contracts. In addition to the experience of teamwork, and the availability of executive technical tools.

The fifth part wraps the social factors to start the PPPs project. Two main questions are required to be answered about the importance of local community support and its acceptance of the project. Moreover, the possibility of providing additional work opportunities that decrease the unemployment rate.

The sixth part is connected to environmental factors. Two questions are presented in the questionnaire about the impact of the project on the surrounding environment.

#### 4.1.2 Exclusive factors

The first part deals with the coordination between the project-related parts. This coordination should be a result of the collaboration of six ministries which are Finance, Local Administration, Foreign Affairs, Social Affairs, Economy and Foreign Trade, and Planning and International Cooperation.

The second part is the project documents part. Eight questions are introduced in this part about the reference presence of the project. That includes the term sheet, purchase and sale documentation, the technical specification document, the feasibility study, and the deadlines.

The third part includes five questions related to the geographical location. Those questions clarify the nearest location to the power transfer lines and also evaluate the safety of the location.

The fourth part is related to the environmental conditions for the project and its social effects. In this part, five questions are introduced for discussion about the soil location and its proximity to residential areas. It also estimates the project's ability to create work opportunities and transfer knowledge and technology to the community.

The fifth part covers the economic side of the PPPs project. Five questions are discussed. The lifetime of the PPPs project is divided into the excellence period, the fixed and flexible budget management and the product service price, and the used electricity distribution investment system. Furthermore, the possibility of increasing the capacity of the power plant because of the power demand increment.

The sixth part deals with the technical side of the project. Six questions are presented including the governmental team experience and the private sector in this project's field. Moreover, the measures of the energy outcomes and their conformity with the technical specifications.

The seventh part is about the legal, management, and organizational parts. Seven questions are clarified in this part which are the private sector's capacity towards the technical dangers, the capacity of the local sector towards the legislations dangers, the capacity of both sectors towards the financial dangers, the project structure, the criteria for choosing the project manager, and the role of the management team in the project and the commitment of the parties towards the contract's conditions.

### 4.2 Statistical analysis

SPSS software has been used to analyze and validate the data. Samples were loaded into SPSS using numerical type for variables, and a five-degree scale of importance. Thereafter, Cronbach's alpha stability coefficient was measured to calculate the degree of stability and validity of the questionnaire samples. The stability was greater than 0.7 which indicates that there are no unclear or abnormal samples, and consequently, the questionnaire is correct and reliable.

Table 1 shows the sample analysis by SPSS for the general factors questionnaire of the PPPs. It is shown from this table that the stability factor is greater than 0.7. Therefore, the general factors questionnaire is correct and it can be included in the study.

**Table 1:** Reliability and validity Coefficients of the general factors questionnaire.

Part	Factor	Validity Coefficient	Reliability Coefficient
1	Commercial and Financial	0.89	0.788
2	Political	0.42	0.179
3	Legal	0.86	0.743
4	Technical	0.73	0.529
5	Social	0.74	0.547
6	Environmental	0.92	0.847

Total	20	0.94	0.883

Table 2 shows the result analysis of the executive factors that affect the PPPs contracts in the electrical energy sector. The stability factor is greater than 0.7 in this table. Thus, the executive factors questionnaire is also valid and it can be included in the study.

**Table 2:** Reliability and validity Coefficients of the executive factors questionnaire.

Part	Factor	Validity Coefficient	Reliability Coefficient
1	Coordination with the project related parties	0.86	0.74
2	Project documents	0.86	0.744
3	Geographical location	0.74	0.548
4	Environmental conditions and social effects	0.80	0.642
5	Economical domain	0.81	0.649
6	Technical part	0.75	0.563
7	Legal, management, and organizational parts	0.76	0.579

Total	42	0.94	0.876

### 4.3 Generative adversarial neural networks (GAN)

The GAN is an unsupervised machine learning technique in which, two neural network models, the generator and discriminator, compete with each other to become more accurate in predictions, after satisfactory training, the generator provides more realistic data without using the discriminator.

The generative model uses one class of objects, the collected data via questionnaire in this case, *Y*, along with noise vector, *ξ*, to generate realistic data with a set of features, *X* as described in (1):

(1) ξ,Y→X

In this work, features are the responses to questions. The noise vector *ξ* is a random vector used to prevent yielding the same *X* always. However, *ξ* is an input to the generator and it is not a learnable vector. On the other hand, the discriminator, or the classifier, uses a set of features to differentiate between real and fake data that are fed to it by the generator (2):

(2) X→Y

The binary cross-entropy (BCE) based loss function which is defined in (3) is used in GAN, because it generates two classes, fake and real.

(3) $$J(\theta )=-\frac{1}{m}\sum_{i=1}^{m}\left[y^{\left(i\right)}\;log\;h(x^{\left(i\right)},\theta )+(1-y^{\left(i\right)})\;log\;(1-h(x^{\left(i\right)},\theta ))\right]$$

Where $-\frac{1}{m}$ is the average loss of the whole batch, *h* represents the prediction parameterized by *θ*, $y^{\left(i\right)}$ is the sample label, and $x^{\left(i\right)}$ is the feature.

**Figure 1:**
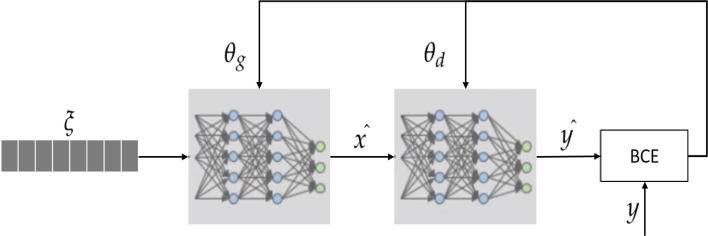
Details of GAN architecture.

The BCE expression has two parts, one relevant to each class. Its values are close to 0 when the label, $y^{\left(i\right)}$, and the prediction are similar. But its value approaches ∞ when the label, $y^{\left(i\right)}$, and the prediction are different. Fig. 1 represents the GAN architecture which has two parts, the generator ($\theta_{g}$) that learns to generate plausible data. The generated instances become negative training examples for the discriminator. The discriminator ($\theta_{d}$) that learns to distinguish the generator's fake data from real data. The noise is input to the generator to produce a featured data, $x^{\overset{ˆ}{}}$. Then using the CNN its weights $\theta_{g}$ are updated based on the positive feedback from the discriminator. The discriminator uses the CNN model to classify $x^{\overset{ˆ}{}}$ as a real or fake data. The CNN weights, $\theta_{d}$, in discriminator are updated based on BCE and the labels, $y^{\left(i\right)}$. $\theta_{g}$ are updated based on only the positive feedback by the discriminator because the generator knows that all data it produces are fake. Therefore, the generator pays attention only when the data class is real. Contrastingly, the discriminator uses both real and fake labels to update its $\theta_{d}$.

The weights are updated using the backpropagation approach. Therefore, different types of activation functions are applied to calculate the output, $a_{i}^{\left[l\right]}$, of each neuron in CNN as declared in (4):

(4) $$a_{i}^{\left[l\right]}=g^{\left[l\right]}\left(z_{i}^{\left[l\right]}\right)$$

Where $z_{i}^{\left[l\right]}$ is calculated using (5):

(5) $$z_{i}^{\left[l\right]}=\sum_{i=0}^{n}\omega_{i}^{\left[l\right]}\;a_{i}^{\left[l\right]}\;+\;b$$

In (5) $\omega_{i}^{\left[l\right]}$ represents the weights from the layer *l*, *b* is the basis weight.

The activation function should be differentiable which enables the back-propagate of the calculated error, also it should be nonlinear to handle nonlinear problems.

But the rectified linear unit (ReLU) activation function, which is defined as in (6), is the most used function in the hidden layers in CNN. The ReLU function is shown in Fig. 3 From this figure, it is observed that when $z^{\left[l\right]}$ is negative, the function output is 0. Therefore, some neurons would be stuck on the same value, consequently, this would stop the learning process which depends on the derivative to get information about how to update the weights. This issue affects not only the next layers to the stuck neurons but also the previous components that are awaiting updates due to the backpropagation. This problem is called the dying ReLU problem. In the present work the leaky ReLU, which is a modification of ReLU, is used to alleviate this issue. The leaky ReLU is defined in (7) and shown in Fig. 4. The term *α* in (7) is a constant and is set to 0.1 in the simulation study.

(6) $$g^{l}\left(z^{\left[l\right]}\right)=max(0,z^{\left[l\right]})$$

(7) $$g^{l}\left(z^{\left[l\right]}\right)=max(\alpha z^{\left[l\right]},z^{\left[l\right]})$$

**Figure 2:**
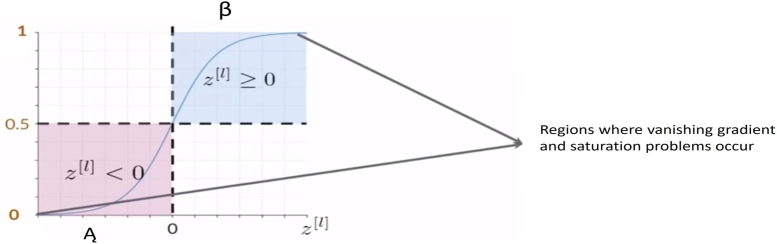
Regions (*A* and *β*) of sigmoid function where vanishing gradient and saturation occur.

The sigmoid activation function defined in (8) is applied only in the last layer of CNN because it suits for nonlinear classification. The sigmoid function is not applied to any of the hidden layers to avoid the vanishing gradient and saturation problems. These problems occur at the tails of the sigmoid function because the derivative of the function approaches zero at these two ends as shown in Fig. 2

(8) $$g^{l}\left(z^{\left[l\right]}\right)=\frac{1}{1+e^{-z^{\left[l\right]}}}$$

**Figure 3:**
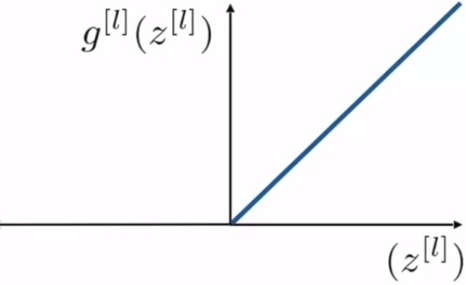
ReLU activation function.

**Figure 4:**
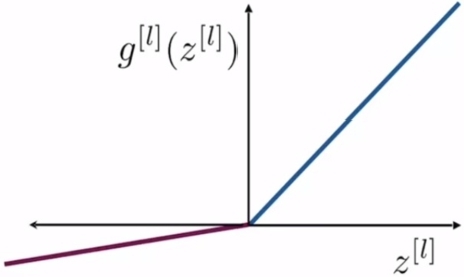
Leaky ReLU activation function.

**Figure 5:**
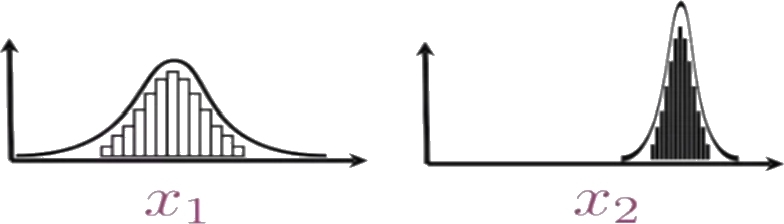
Different distributions of two features in the input layer.

Feature distributions in the input layer of the model are usually different as shown in Fig. 5. This difference causes differences in the weight updates which in turn affects the cost function and slows down the learning. Therefore, it is vital to normalize $z_{i}^{\left[l\right]}$ in each neuron. Normalization provides faster and easier learning and helps to avoid the covariate shift problem which happens when the distributions of training and testing data for some features are different. The normalized ${z^{\overset{ˆ}{}}}_{i}^{\left[l\right]}$ is calculated in the training phase using (9):

(9) $${z^{\overset{ˆ}{}}}_{i}^{\left[l\right]}=\frac{z_{i}^{\left[l\right]}-\mu_{z_{i}^{\left[l\right]}}}{\sqrt{\sigma_{z_{i}^{\left[l\right]}}^{2}+\epsilon }}$$

Where $\mu_{z_{i}^{\left[l\right]}}$, $\sigma_{z_{i}^{\left[l\right]}}^{2}$ is the mean and the standard variation respectively. *ϵ* is a small number to prevent the denominator from being zero. In the testing phase, ${z^{\overset{ˆ}{}}}_{i}^{\left[l\right]}$ is computed as in (10):

(10) $${z^{\overset{ˆ}{}}}_{i}^{\left[l\right]}=\frac{z_{i}^{\left[l\right]}-E(z_{i}^{\left[l\right]})}{\sqrt{Var(z_{i}^{\left[l\right]})+\epsilon }}$$

Where $E(z_{i}^{\left[l\right]})$ is the expected value of $z_{i}^{\left[l\right]}$, and $Var(z_{i}^{\left[l\right]})$ is the standard variation of $z_{i}^{\left[l\right]}$. The output $y_{i}^{\left[l\right]}$ is then computed according to equation (11):

(11) $$y_{i}^{\left[l\right]}=\gamma \;{z^{\overset{ˆ}{}}}_{i}^{\left[l\right]}+\beta$$

Where γ,β represent the scale and shift factors. They are learnable parameters to obtain the optimal distribution. Normalization smooths the cost function and reduces the internal covariate shift.

### 4.4 Analytic Hierarchy Process (AHP) theory analysis by using expert choice software

AHP theory is employed in this work to calculate the every factor of both general and executive criteria in the PPPs scheme. AHP is powerful in providing organized solutions to decision-makers. Those solutions are provided by dividing the problem into smaller parts and then sorting those parts into levels according to their importance by using Expert Choice software. This software is able to provide the possibility of extracting suitable result schedules, executing and designing graphic design for the pyramid, and organizing the priorities and preferences. After collecting the samples and validating them using SPSS, AHP tool is used to organize the criteria in relation to each other depending on weights calculated by the software itself based on the binary comparison. The criteria with low weights can be neglected and that helps decision-makers in their strategies.

AHP first determines the problem statement and goals. Then, it builds a decision structure with a pyramid shape. The main goal locates at the top, the criteria at the middle, and the other choices at the bottom of the pyramid. A set of pairwise comparison matrices are further created and each member from the upper level is compared with the other elements in the direct lower level. After made all the pairwise operations, the consistency ratio (*CR*) is calculated using (12) depending on the stability index (*CI*) and the liner transforming coefficient (*λ*):

(12) $$CI=\frac{\lambda -n}{n-1}$$

Where *n* is the matrix size. *CR* factor is then calculated using the relation in (13):

(13) $$CR=\frac{CI}{RI}$$

Where *RI* is a random stability index. Thereafter, the software is used to input the requested main and secondary criteria according to their weights. Then, enter the number of the participating experts in the questions of the questionnaire along with answers to the questionnaire for the general and the exclusive criteria. Results are collected from the AHP tool and further discussed in the next section.

## 5 Experimental results and discussion

Table 3 lists the weights for general factors of PPPs contract that are calculated by AHP. It is observed from this table that

**Table 3:** Weights for the general factors in PPPs.

Factor	Weight (%)
Political	29.7
Legal	16.1
Social	16
Commercial and Financial	15.5
Technical	11.5
Environmental	11.2

The political factor is the most important one in PPPs in Syria. That is because the ongoing war affects the PPPs economically in Syria due to the non-stability. All countries that employ PPPs contracts successfully have legal structures and special guides for each partnership contract. That minimizes the possibility of having problems that follow this type of contract. Therefore, the legal criteria occupy the second important factor. Act No. 5 in 2016 regarding partnership contracts ensures the importance of legal criteria and specifies the rights and obligations to put the project in the correct direction. The social factor is classified third because of the importance of society and its prior acceptance of this type of contract.

The costs of establishing power plants represent a significantly heavy load on the government's shoulders that gives the private sector present and economical returns benefits. Furthermore, decreasing the financial expenses supported by the government is a vital criterion in partnership contracts. The technical criteria have the fifth importance order because it is crucial for both sectors to start the project professionally. PPPs contracts provide modern techniques which help to transfer knowledge to a bigger group of workers. Furthermore, the environmental criteria are also important considering sustainability and preserving the environment.

Table 4 lists the weights for exclusive factors of PPPs contract. It is observed from this table that the 42 exclusive criteria are grouped into 7 parts as follows

**Table 4:** Weight for the exclusive factors in PPPs.

Part	Question	Factor	Weight (%)
**Table:** Coordination between parties	1	Role of the finance ministry in providing the guarantees and exemptions	1.4136
	2	Role of planning and international cooperation in providing needed support and advice	0.931
	3	Role of the ministry of local administration in issuing licenses regarding the environmental impact assessment, and the expropriation procedures necessary to pass the power transmission lines	1.3623
	4	Role of the ministry of foreign affairs and expatriates in advertising the project abroad	0.9614
	5	Role of the ministry of social affairs and labor in granting work permits to foreign staff	0.8246
	6	Role of the ministry of economy and foreign trade in granting licenses to import the required equipment and supplies	1.3623

Project documents	7	Existence of reference vision and preliminary project document	2.863
	8	Existence of term sheet and clear evaluation criteria	3.031
	9	Management and organization operations for project's data	2.527
	10	Existence of procedure guide	2.254
	11	Documentation for purchases and sales	2.464
	12	Existence of clear criteria for the handover and receiving process after the excellence period finishes	2.737
	13	Accuracy and reliability of the technical studies	3.031
	14	Existence of economic feasibility	3.176

**Table:** Geographical location	15	The location's proximity to power transition lines and the main roads	2.3024
	16	Location's proximity to power transition lines	2.2274
	17	Location's safety and its distance from border areas	1.869
	18	Availability of water resources	2.261
	19	Distance to the city center and commute costs	1.3986

**Table:** Environmental conditions	20	Soil testing for construction	1.699
	21	Distance from residential places	2.4952
	22	Employment opportunities	2.0354
	23	Subcontracting works	1.5598
	24	Knowledge transfer	2.163

**Table:** Economic domain	25	Excellence period's effect on the private sector	3.5738
	26	Changing and fixed cost management that reduces the production cost for MW/h	3.6106
	27	Price of the service provided	4.2178
	28	Electricity distribution investment system	3.3346
	29	Possibility of increasing the capacity of the plant	4.3472

Technical side	30	Domain experts from the government end	1.408
	31	Specifications for produced energy outcomes	1.476
	32	Professional, technical, administrative, structural, and organizational qualifications of partners	1.6715
	33	Quality of service	1.7755
	34	Matching specifications and the technical conditions	1.595
	35	Training provided by the private sector during the contractual period to transfer the experiences to the government employees	1.391

Administration sides	36	Ability of the private sector of managing technical risks	2.737
	37	Ability of the private sector of managing legislative risks	2.8455
	38	Ability of partners to bear financial risks caused by increased prices and inflation	3.4965
	39	Project structure	2.7153
	40	Project managers and sub-teams mangers seniority	3.4097
	41	Communication between consultants and partners	2.8889
	42	Commitment toward the contract terms	4.5571

The coordination with the project's related parties through considering 6 criteria. The finance ministry has the first priority in this part because of its responsibility to provide the necessary guarantees and exemptions for PPPs. In addition to the flexibility of the tax relief that motivates the private sector to invest safely. The project documents including the eight criteria are introduced. The feasibility study has the highest priority because it gives a clear vision of revenues for both sectors in PPPs and in other types of projects scheme as well. The technical studies' accuracy and reliability decrease the technical risks during the execution. Its reliability is important in PPPs contracts to ensure delivery conditions. The existence of a term sheet, solicitation documents, and clear evaluation standards due to their importance in settling bids and, consequently, choosing the most suitable partner.

The geographic location. Five criteria are discussed and the locations' proximity to the power transportation has the first priority. Energy transportation and land acquisition are always considered among the difficult execution factors. In Rastin city, There is more than 20 KM required land to be acquired for the power plant. Another factor is the availability of water resources and water storage tanks and energy resources. The environmental conditions of the PPPs project and its social impact have five measures. The locations' distance from the residential places has primacy because the closer distance increases the stability factors of the electrical system. Transferring knowledge and technology and placing them in the community is also an important factor in PPPs. Transferring operations of the technical knowledge and executing technology is extremely critical when the partner is foreign. For example, the Rastin power plant project includes foreign experts from MAPNA group which is a leading company in the power plants industry in the middle east.

The economic domain of the PPPs project has five criteria. The possibility of increasing the capacity of the plant upon increased demand for energy has the first priority in this part. The next factor is the suggested service price because of its direct effects on the community for accepting the project. Therefore, the price should be suitable for local wages. The third factor of priority in this part is fixed and changeable cost management. Should changeable costs were managed successfully, prices for the electricity would be fixed regardless of the MW/h production cost. The next factor is the duration of the excellence period. Long excellence periods motivate investors and promise them to have valuable revenue. The technical part of the project has six criteria and service quality has the first priority among them. The private sector should guarantee service quality in order to gain the surrounding community's acceptance. The next criteria are the technical, skillful, administration, structural, and organizational qualifications of the partner. Those qualifications should be considered to evaluate the ability of the private sector to excel in this type of project.

The legal, administrative, and organizational sides include seven criteria. The commitment of the contractual parties to the PPPs contract provisions has the first priority in this part. The second factor of importance is the capacity of both sectors to manage financial risks. Those risks come from expensiveness and inflation within the excellence period. Therefore, it is necessary to evaluate the capability of each sector to take the risks.

## 6 Conclusion and policy recommendations

The use of Public-Private Partnerships (PPPs) in developing countries like Syria is crucial in order to avoid excessive costs for the government and promote sustainable development goals. The involvement of the private sector is essential in the development process, but it is important to carefully define collaborations between the private and public sectors. To aid in this decision-making process, a comprehensive decision support system is recommended. Collecting data for this study was challenging due to the crisis in Syria and its status as a developing country. As a result, we relied on questionnaires based on published studies and interviews with experts in the field. The limited sample size was a drawback, but the use of artificial intelligence (AI) and generative adversarial networks (GANs) helped to strengthen the data and provide decision-makers with reliable information. The experimental results regarding the use of PPPs in the Syrian electrical system highlighted several important criteria, including political, legal, social, economic, technical, and environmental factors. These criteria directly impact partnership contracts for electrical projects. Understanding which criteria are indispensable and which can be disregarded is crucial for decision-makers when making informed choices. The case study presented in this research reflects the current state of affairs in Syria. We recommend adhering to PPP legislation for service projects to alleviate the financial burden on the government. Additionally, it is important to review the general criteria for this type of contract and study exclusive criteria based on the project type, ensuring that partnership decisions are not made randomly. This study can be expanded to include more criteria in both general and exclusive aspects. Moreover, employing artificial intelligence models can help facilitate smarter and more accurate weighting calculations.

## CRediT authorship contribution statement

**Vladimír Krepl:** Conceptualization, Formal analysis, Investigation, Methodology, Writing – original draft, Writing – review & editing. **Ghaeth Fandi:** Conceptualization, Formal analysis, Funding acquisition, Investigation, Methodology, Project administration, Software, Supervision, Validation, Writing – original draft, Writing – review & editing. **Mohammad Rehabi:** Data curation, Methodology, Resources, Software, Validation, Visualization, Writing – original draft, Writing – review & editing. **Safwan Ghanem:** Conceptualization, Data curation, Formal analysis, Investigation, Methodology, Resources, Software, Validation, Visualization, Writing – original draft, Writing – review & editing. **Fayez Jrad:** Conceptualization, Data curation, Formal analysis, Funding acquisition, Investigation, Methodology, Project administration, Resources, Software, Supervision, Writing – original draft. **Zdenek Muller:** Conceptualization, Funding acquisition, Investigation, Methodology, Project administration, Resources, Supervision, Validation, Writing – review & editing. **Luboš Smutka:** Conceptualization, Funding acquisition, Investigation, Methodology, Project administration, Software, Supervision, Validation, Writing – original draft. **Jan Kyncl:** Conceptualization, Formal analysis, Investigation, Methodology, Visualization, Writing – review & editing. **Melkior Urbanus:** Conceptualization, Data curation, Investigation, Methodology, Visualization. **Soliman Fandie:** Conceptualization, Formal analysis, Investigation, Software. **Inna Čábelková:** Conceptualization, Data curation, Investigation, Supervision, Writing – original draft. **Josef Tlustý:** Formal analysis, Investigation, Methodology, Resources, Writing – original draft.

## Declaration of Competing Interest

The authors declare that they have no known competing financial interests or personal relationships that could have appeared to influence the work reported in this paper.

## Data Availability

Data will be made available on request.
